# A rapid and accurate guanidine CEST imaging in ischemic stroke using a machine learning approach

**DOI:** 10.1088/1361-6560/ae4167

**Published:** 2026-02-18

**Authors:** Malvika Viswanathan, Leqi Yin, Yashwant Kurmi, You Chen, Xiaoyu Jiang, Junzhong Xu, Aqeela Afzal, Zhongliang Zu

**Affiliations:** 1 Vanderbilt University Institute of Imaging Science, Vanderbilt University Medical Center, Nashville, TN, United States of America; 2 Department of Biomedical Engineering, Vanderbilt University, Nashville, TN, United States of America; 3 School of Engineering, Vanderbilt University, Nashville, TN, United States of America; 4 Department of Radiology and Radiological Sciences, Vanderbilt University Medical Center, Nashville, TN, United States of America; 5 Department of Biomedical Informatics, Vanderbilt University Medical Center, Nashville, TN, United States of America; 6 Department of Computer Science, Vanderbilt University, Nashville, TN, United States of America; 7 Department of Physics and Astronomy, Vanderbilt University, Nashville, TN, United States of America; 8 Department of Neurological Surgery, Vanderbilt University Medical Center, Nashville, TN, United States of America

**Keywords:** chemical exchange saturation transfer (CEST), guanidine CEST imaging, machine learning, ischemic stroke

## Abstract

*Objective.* Rapid and accurate mapping of brain tissue pH is crucial for early diagnosis and management of ischemic stroke. Amide proton transfer (APT) imaging has been used for this purpose but suffers from hypointense contrast and low signal intensity in lesions. Guanidine chemical exchange saturation transfer (CEST) imaging provides hyperintense contrast and higher signal intensity in lesions at appropriate saturation power, making it a promising complementary approach. However, quantifying the guanidine CEST effect remains challenging due to its proximity to water resonance and the influence of multiple confounding effects. This study presents a machine learning (ML) framework to improve the accuracy and robustness of guanidine CEST quantification with reduced scan time. *Approach.* The model was trained on *partially synthetic data*, where measured line-shape information from experiments were incorporated into a simulation framework along with other CEST pools whose solute fraction (*f*_s_), exchange rate (*k*_sw_), and relaxation parameters were systematically varied. Gradient-based feature selection was used to identify the most informative frequency offsets to reduce the number of acquisition points. *Main results.* The proposed model achieved significantly higher accuracy than polynomial fitting, multi-pool Lorentzian fitting, and ML models trained solely on synthetic or *in vivo* data. Gradient-based feature selection identified the most informative frequency offsets, reducing acquisition points from 69 to 19, a 72% reduction in CEST scan time without loss of accuracy. *In vivo*, conventional fitting methods produced unclear lesion contrast, whereas our model predicted clear hyperintense lesion maps. The strong negative correlation between guanidine and APT effects supports its physiological relevance to tissue acidosis. *Significance.* The use of partially synthetic training data combines realistic spectral features with known ground-truth values, overcoming limitations of purely synthetic or limited *in vivo* datasets. Leveraging this data with ML, enables robust quantification of guanidine CEST effects, showing potential for rapid pH-sensitive imaging.

## 1. Introduction and objective

Acute ischemic stroke is a major cause of death and disability worldwide, posing considerable challenges of timely intervention (Benjamin *et al* 2019). The primary objective of acute ischemic stroke therapy is to preserve tissues at-risk through reperfusion. Current treatment methods are limited by a narrow therapeutic window of 4.5 h. However, recent advances have underscored the critical role of imaging in extending the eligibility for endovascular therapy (EVT) to late-presenting stroke patients (Nogueira *et al* 2018, Albers *et al* 2018, Olthuis *et al* 2023, Kobeissi *et al* 2023). These studies suggest that imaging-based assessments, which focus on identifying viable brain tissue (i.e. penumbra, Hossmann 1994), offer an effective approach to select patients who may benefit from EVT rather than traditional time constraints.

Ischemic stroke triggers anaerobic metabolism and lactate production, leading to a drop in tissue pH. Previous studies have suggested that pH-sensitive imaging holds significant potential for identifying salvageable tissue in the penumbra, thereby improving stroke management (Hossmann 1994, Sun *et al* 2007, Zhou and van Zijl 2011, Harston *et al* 2015). An emerging MRI contrast mechanism, chemical exchange saturation transfer (CEST), detects solute molecules with exchangeable protons and provides information about their chemical environments, such as pH levels (Ward *et al* 2000, Zhou and van Zijl 2006, van Zijl and Yadav 2011, Jin *et al* 2013, 2024, Liu *et al* 2013, Kim *et al* 2015, Wu *et al* 2016, van Zijl *et al* 2018, Vinogradov 2019, Jin and Chung 2022, Chung *et al* 2024). In CEST imaging, solute protons are initially saturated using a long, frequency-selective RF saturation pulse. This saturation is then transferred to the bulk water protons through chemical exchange, resulting in a significant reduction in water magnetization. By measuring the changes in the water signal, valuable information reflecting both solute concentration and tissue pH can be indirectly obtained with enhanced detection sensitivity.

In biological tissues, amide proton transfer (APT) is a major CEST effect at approximately 3.5 ppm from water, originating from the backbone amide of mobile proteins/peptides (Zhou *et al* 2003). Previous studies have demonstrated that the APT signal is pH-sensitive and holds promise for detecting the ischemic penumbra (Sun *et al* 2007, 2011b, Tee *et al* 2014, Tietze *et al* 2014). However, the amide–water exchange rate is in the slow exchange regime, resulting in a reduced APT signal intensity in stroke lesions under commonly used saturation fields (e.g. 0.5–1.25 *μ*T) in stroke-related applications (Zhou *et al* 2003, Sun *et al* 2007, 2010, 2011a, 2011b, 2012, Jin *et al* 2013, Tee *et al* 2014, Tietze *et al* 2014, Harston *et al* 2015, Msayib *et al* 2019, Wang *et al* 2019). Guanidine CEST is another major CEST effect at around 2 ppm (Cai *et al* 2015a) which, in the brain, arises from both protein arginine and creatine (Chen *et al* 2017, Zhang *et al* 2017b). The guanidine-water exchange rate falls within the slow-intermediate exchange regime (Haris *et al* 2014, Kogan *et al* 2014, Rerich *et al* 2015, Cai *et al* 2015a, Wu *et al* 2018, Wu and Sun 2023, Zhang *et al* 2023). Consequently, under commonly used saturation powers, guanidine CEST can provide an enhanced signal intensity in stroke lesions, offering hyperintense contrast as a potential advantage over APT imaging in enhancing lesion visibility. Simulations in supporting information figure S1 illustrate the contrasting dependence of the APT and guanidine CEST effects on *k*_sw_.

However, accurate and specific quantification of the guanidine CEST at 2 ppm presents more challenges compared to APT at 3.5 ppm due to two key factors. First, there is a more pronounced direct water saturation (DS) effect at 2 ppm, which overlaps with the guanidine CEST. Second, the presence of fast-exchange amine CEST effect, which typically resonates at around 3 ppm, shifts closer to the water peak due to the coalescence effect, thus exerting a more substantial influence at 2 ppm (Cai *et al* 2012, Zhang *et al* 2017a, 2018). Polynomial fit-based methods have been used to quantify the guanidine CEST (Chen *et al* 2017, 2019). However, in polynomial fitting, the selection of reference signal and the order of polynomial coefficients may lead to either overestimation or underestimation of the guanidine CEST effect. Alternatively, multiple-pool model Lorentzian fitting methods have been employed to resolve various CEST effects. However, multiple-pool model Lorentzian fit, which relies on nonlinear least-squares fitting, is prone to local minima due to its sensitivity to initial values and noise, especially as the number of overlapping pools increases. Our recent study indicates that this approach may not effectively isolate the guanidine CEST effect from the rapidly exchanging amine CEST effect (Cui *et al* 2022).

Machine learning (ML) revolutionizes data analysis by efficiently uncovering hidden patterns and trends that traditional methods often miss. It excels at extracting meaningful insights from complex datasets, revealing information that would otherwise remain obscured. Additionally, ML can automatically identify and select the most useful features by calculating their importance, thereby optimizing the input data (Li *et al* 2020, Bie *et al* 2022, Cheema *et al* 2024, Cheema *et al* 2025, Shen *et al* 2025). This optimization can lead to a reduction in acquisition time for CEST imaging, which is critical for the application in acute stroke. Previously, ML has been successfully applied to improve the quantification of the APT effect (Glang *et al* 2020, Kim *et al* 2020, Huang *et al* 2022). To effectively train ML neural network (NN) models for predicting the APT effect, our previous work introduced an innovative platform for generating synthetic CEST signals by integrating APT, various other CEST effects, DS, and magnetization transfer (MT) effects using an inverse summation relationship (Zaiss *et al* 2015), rather than using the Bloch equation simulations. This approach allows the use of these effects from either signal model calculations or Lorentzian fitting of measured data to synthetize CEST signals, thereby producing what is referred to as partially synthetic data (Viswanathan *et al* 2024, 2025a). This method is more practical for training NN models since it effectively addresses the challenges associated with limited data availability and the lack of high-quality ground truth data by using measured data for training. It also provides data with better fidelity than the fully synthetic data simulated from Bloch equations, which require accurate setting of the number of simulation pools and the range of their concentration, exchange, and relaxation parameters. Specifically, the line shape of the background signal near 3.5 ppm is vital for ML prediction of the APT effect. However, this background signal results from multiple amines and potentially other pools (Zong *et al* 2014). Its line shape information near 3.5 ppm cannot be accurately replicated by simple model simulations but can be more precisely measured using a multiple-pool model Lorentzian fit of a single pool centered within 2–3 ppm, which reflects a combined contribution from amines and guanidine, excluding the APT. While the model trained on data generated using this method effectively predicts APT, it cannot be directly used to quantify the guanidine CEST effect due to its significant overlap with the amine CEST effect. In this study, we improve this platform by employing a polynomial fit to further process the multiple-pool model Lorentzian fitted pool centered within 2–3 ppm to provide a more accurate estimation of the line shape information of the background signal related to the guanidine CEST effect. This enables the generation of partially synthetic data, incorporating diverse variations in the guanidine CEST effect and its background signals, enabling the training of an ML network to accurately predict the guanidine CEST effect. Additionally, we optimized the acquisition data to shorten the total scan time using our ML method. We began by validating our method’s accuracy against traditional CEST quantification techniques, using ground truth provided from simulations of tissue mimicking data. This approach was then applied to animal models of ischemic stroke, where its performance was compared to both conventional methods and ML networks trained on *in vivo* and fully synthetic data. Additionally, we demonstrated its potential for pH imaging by correlating the results with APT effect.

## 2. Approach

### 2.1. Animal preparation

A middle cerebral artery occlusion (MCAO) method was employed to induce ischemic strokes in five rat brains. A 30 mm silicon-coated 4-0 nylon suture (Doccol Corporation, Redlands, CA, USA) was inserted into the left internal carotid artery and advanced to a depth of 18–20 mm to occlude the MCA. The ischemic stroke was induced by tightening the suture around the filament, followed by sealing the incision. Post-surgery, the animals were immediately moved to the MRI scanner. During the surgery and imaging, the animals were anesthetized with 2%–2.5% isoflurane, their respiration was monitored, and their body temperature was kept at 37 °C using a warm air circulation system. All procedures were approved by the Vanderbilt University Medical Center Institutional Animal Care and Use Committee *(M12048 approved 06/05/2012 for stroke induction and M2000039-01 approved 05/10/2023 for all other procedures)*.

### 2.2. MRI

Experiments were conducted on a Varian DirectDrive™ horizontal 7T MRI system equipped with a 38 mm Doty RF coil (Doty Scientific Inc., Columbia, SC, USA). Baseline images were scanned 1–3 d prior to MCAO surgery. On the day of surgery, images were acquired at post-surgery time intervals of 0.5–1 h, 1–1.5 h, and 1.5–2 h. CEST imaging utilized a sequence comprising of a 5 s continuous wave (CW) RF saturation pulse with minimum TE, followed by a 2 s recovery period (TR = 7 s). Single-shot spin-echo echo planar imaging with one average was used to acquire CEST data. Frequency offsets ranging from −13.33 ppm to 13.33 ppm (corresponding to Δ*ω* values of ±4000 Hz, ±3500 Hz, ±3000 Hz, ±2500 Hz, −1500:50:1500 Hz) were used to acquire Z-spectral signals (S) while control signals (*S*_0_) were acquired at 333 ppm (corresponding to Δ*ω* of 100 000 Hz). All CEST signals were acquired at a saturation field strength (*B*_1_) of 1 *μ*T. A selective inversion recovery quantitative magnetization transfer (qMT) technique (Gochberg and Gore 2007) with four averages was utilized for assessing the observed water longitudinal relaxation time (*T*_1obs_ = 1/*R*_1obs_) and MT pool size ratio (*f*_m_). The apparent diffusion coefficient (ADC) data were collected using a pulse gradient spin-echo sequence with simultaneous gradients applied along three axes. The sequence featured a gradient duration of 6 ms, a separation time of 12 ms, and five b-values ranging between 0 and 1000s mm^−2^ and one average. The matrix size was 64 × 64 and the field of view was 30 mm × 30 mm for all scans. It took approximately 8 mins, 2.5 mins, and 31 s to acquire an entire CEST Z-spectrum, qMT, and ADC data respectively.

### 2.3. CEST quantification metric

To accurately quantify a target CEST effect from the acquired CEST signals, also known as the label signal (*S*_lab_), it is essential to obtain a reference signal (*S*_ref_) that encompasses all contributions except for the target CEST effect. To enhance the specificity of this quantification, we utilized an inversion subtraction of the label and reference signals combined with *T*_1obs_ normalization, instead of the conventional direct subtraction method. This approach is referred to as the apparent exchange-dependent relaxation (AREX) metric (Zaiss et al 2015), which is defined as:

\begin{align*}{\mathrm{AREX}}\left( {\Delta \omega } \right) = \left( {\frac{{{S_0}}}{{{S_{{\mathrm{lab}}}}\left( {\Delta \omega } \right)}} - \frac{{{S_0}}}{{{S_{{\mathrm{ref}}}}\left( {\Delta \omega } \right)}}} \right){R_{{\mathrm{1obs}}}}\left( {1 + {f_{\mathrm{m}}}} \right) = {R_{{\mathrm{ex}}}}\left( {\Delta \omega } \right)\end{align*}

where *R*_ex_ represents the CEST effect in the rotating frame, specifically reflecting the solute–water exchange effect. This can be described by Zaiss and Bachert (2013)

\begin{align*}{R_{{\mathrm{ex}}}}\left( {\Delta \omega } \right) = {\text{ }}\frac{{{f_{\mathrm{s}}}{k_{{\mathrm{sw}}}}\omega _1^2}}{{\omega _1^2 + \left( {{R_{{\mathrm{2s}}}} + {k_{{\mathrm{sw}}}}} \right){k_{{\mathrm{sw}}}} + \frac{{{{\left( {\Delta \omega - \Delta } \right)}^2}{k_{{\mathrm{sw}}}}}}{{{R_{{\mathrm{2s}}}} + {k_{{\mathrm{sw}}}}}}}}\end{align*}

where *f*_s_, *k*_sw_ and *R*_2s_ represent the solute concentration, solute–water exchange rate and solute transverse relaxation rate. ${\omega _1} = \gamma {B_1}$, where $\gamma $ is the gyromagnetic ratio of proton. $\Delta \omega $ represents the list of frequency offsets. $\Delta $ represents the solute central frequency offset from water.

### 2.4. CEST analysis methods

A three-point (3pt) method (Jin *et al* 2013, Xu *et al* 2014), which uses the average of CEST signals at 3 ppm and 4 ppm to obtain *S*_ref_ and the CEST signal at 3.5 ppm as *S*_lab_, has been previously employed to quantify the APT effect. While this method may underestimate the APT effect, it minimizes contributions from all confounding factors. In this study, we utilized the three-point method for APT quantification. *R*_ex_ for the APT effect from this three-point method was quantified using the AREX metric and termed AREX_3pt_APT_. A polynomial and Lorentzian line-shape fitting (PLOF) (Chen *et al* 2017, 2019) and multiple-pool model Lorentzian fit (mfit) (Cai *et al* 2015a, Zhang *et al* 2016, Tee *et al* 2017, Wu *et al* 2018) have been used to quantify the guanidine CEST effect. In the PLOF method, the guanidine CEST effect was modeled by a Lorentzian function, while the background signals, which include broad amine CEST and MT effects, were modeled by a polynomial function. Specifically, a third-order polynomial function, similar to that used in previous papers (Chen *et al* 2017, 2019), was employed. The CEST Z-spectrum was modeled as an inverse summation of all these CEST effects, the MT effect, and the water relaxation effect. *R*_ex_ for the guanidine CEST effect ($R_{{\mathrm{ex}}}^{{\mathrm{guan}}}$) was obtained by fitting the Z-spectrum to this inverse summation function, termed AREX_PLOF_. In the multiple-pool model Lorentzian fit method, each pool was modeled by a Lorentzian function, and the 1—Z-spectrum was modeled by the sum of all these Lorentzian functions. Each Lorentzian was individually fitted, and *S*_ref_ was obtained by reconstructing the Z-spectrum with the amplitude of the fitted target Lorentzian set to zero. *S*_lab_ was taken from the measured CEST signals. A six-pool model Lorentzian fit incorporating amide, amines/guanidine, water, NOE at −1.6 ppm (referred as NOE (−1.6)), NOE at −3.5 ppm (referred as NOE (−3.5)) as well as MT was utilized. $R_{{\mathrm{ex}}}^{{\mathrm{amines/guan}}}$ was fitted from the six-pool model Lorentzian fit and quantified using the AREX metric, termed AREX_mfit_6pool_. Additionally, a seven-pool model Lorentzian fit was utilized, resolving amines and guanidine into separate pools. The $R_{{\mathrm{ex}}}^{{\mathrm{guan}}}$ effect obtained from the seven-pool model Lorentzian fit and quantified using the AREX metric was termed AREX_mfit_7pool_. Lastly, a third-order polynomial fit was applied to quantify the guanidine CEST effect by fitting the measured background CEST signals between 1–1.5 ppm and 2.5–3 ppm to obtain *S*_ref_, following the method described in a previous paper (Cui *et al* 2022). The $R_{{\mathrm{ex}}}^{{\mathrm{guan}}}$ effect obtained using the polynomial (poly) fit and quantified with the AREX metric was termed AREX_poly_. The fitting aimed to minimize the root mean square of residuals between measured data and the model. Supporting information tables S1–S4 lists the initial and boundary conditions for the PLOF, the six-pool and seven-pool model Lorentzian fits and the polynomial fit.

### 2.5. Generation of partially synthetic data for training ML model

**Figure 1.**
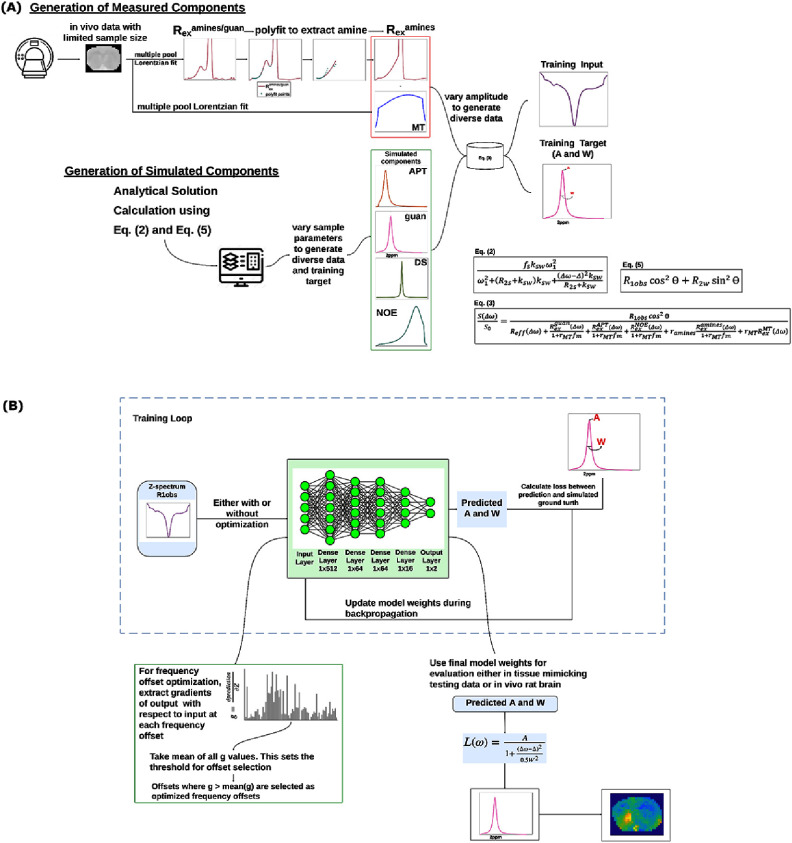
(A) Flowchart describing the process of generating partially synthetic CEST data with simulated and measured components. (B) Flowchart describing the ML workflow, the architecture of the NN model implemented and frequency offset optimization selection.

Figure 1(A) illustrates the workflow used to generate the partially synthetic CEST data. In line with our previous work, we divided all underlying CEST pools or effects into simulated components and measured components (Viswanathan *et al* 2024, 2025a). The simulated components include pools or effects that have simple signal models or with well-defined sample parameter ranges. The measured components include pools that have contributions from multiple overlapping pools or have unknown sample parameter ranges. The partially synthetic CEST data is obtained by an inverse summation of these components, as shown in equation (3),

\begin{align*}\frac{{S\left( {\Delta \omega } \right)}}{{{S_0}}} = \frac{{{R_{{\mathrm{1obs}}}}{{\cos }^2}{{\theta }}}}{{{R_{{\mathrm{eff}}}}\left( {\Delta \omega } \right) + \frac{{R_{{\mathrm{ex}}}^{{\mathrm{guan}}}\left( {\Delta \omega } \right)}}{{1 + {r_{{\mathrm{MT}}}}{f_{\mathrm{m}}}}} + \frac{{R_{{\mathrm{ex}}}^{{\mathrm{APT}}}\left( {\Delta \omega } \right)}}{{1 + {r_{{\mathrm{MT}}}}{f_{\mathrm{m}}}}} + \frac{{R_{{\mathrm{ex}}}^{{\mathrm{NOE}}}\left( {\Delta \omega } \right)}}{{1 + {r_{{\mathrm{MT}}}}{f_{\mathrm{m}}}}} + {r_{{\mathrm{amines}}}}\frac{{R_{{\mathrm{ex}}}^{{\mathrm{amines}}}\left( {\Delta \omega } \right)}}{{1 + {r_{{\mathrm{MT}}}}{f_{\mathrm{m}}}}} + {r_{{\mathrm{MT}}}}R_{{\mathrm{ex}}}^{{\mathrm{MT}}}\left( {\Delta \omega } \right)}}\end{align*}

where $R_{{\mathrm{ex}}}^{{\mathrm{guan}}}$, $R_{{\mathrm{ex}}}^{{\mathrm{APT}}}$, and $R_{{\mathrm{ex}}}^{{\mathrm{NOE}}}$ are simulated components derived using equation (2). $R_{{\mathrm{ex}}}^{{\mathrm{MT}}}$ is a measured component derived from the six-pool model Lorentzian fit, which can be calculated using *R*_1obs_*L*_MT_/(1-*L*_MT_, Cui *et al* 2023), where *L*_MT_ represents the direct subtraction of *S*_lab_ from *S*_ref_ for the MT effect within the Lorentzian fit. $R_{{\mathrm{ex}}}^{{\mathrm{amines}}}$ is another measured component obtained through the combined use of the six-pool model Lorentzian fit and the polynomial method, referred to as mfit-poly. In the mfit-poly approach, $R_{{\mathrm{ex}}}^{{\mathrm{amines/guan}}}$, which has contributions from both amines and guanidine, was initially obtained from the six-pool model Lorentzian fit. Subsequently, $R_{{\mathrm{ex}}}^{{\mathrm{amines}}}$ was separated from $R_{{\mathrm{ex}}}^{{\mathrm{guan}}}$ by fitting $R_{{\mathrm{ex}}}^{{\mathrm{amines/guan}}}$ between 1–1.5 ppm and 2.5–5 ppm to a polynomial function model with coefficients (*C*_0_–*C*_3_). Equation (4) gives the mfit-poly model function,

\begin{align*}R_{{\mathrm{ex}}}^{{\mathrm{amines}}} = {C_0} + {C_1}\left( {\Delta \omega - {\Delta _{{\mathrm{amine}}}}} \right) + {C_2}{\left( {\Delta \omega - {\Delta _{{\mathrm{amine}}}}} \right)^2} + {C_3}{\left( {\Delta \omega - {\Delta _{{\mathrm{amine}}}}} \right)^3}\end{align*}

where ${\Delta _{{\mathrm{amine}}}} = 3\;{\mathrm{ppm}}$. The starting points and fit boundaries for the mfit-poly method are listed in supporting information table S5. Notably, our approach differs from the previous use of PLOF (Chen *et al* 2017, 2019), where the signals from both the amines and MT were modeled as a single polynomial function. In this approach, we separate the amines and MT to adjust their contributions independently. This allows for the generation of diverse datasets with variations in each pool, enhancing the precision and flexibility of the model. The measured components were utilized to provide the line shape information, thereby eliminating the need to know the exact number of overlapping pools or their sample parameter ranges. To tune the amplitude of each measured component, a scaling factor ($r$) was employed. This approach allows for accurate representation of the measured components without requiring detailed knowledge of the underlying pools. *R*_eff_ represents the water relaxations in the rotating frame, which was calculated by,

\begin{align*}\begin{gathered} {R_{{\mathrm{eff}}}}\left( {\Delta \omega } \right) = {R_{{\mathrm{1obs}}}}{\cos ^2}{{\theta }} + {R_{{\mathrm{2w}}}}{\sin ^2}{{\theta }} \hfill \\ {\cos ^2}{{\theta }} = \frac{{\Delta {\omega ^2}}}{{\omega _1^2 + \Delta {\omega ^2}}};\,{\sin ^2}{{\theta }} = \frac{{\omega _1^2}}{{\omega _1^2 + \Delta {\omega ^2}}} \hfill \\ \end{gathered} \end{align*}

where *R*_1obs_ was calculated using (*R*_1w_ + *r*_MT_*f*_m_*R*_1M_)/(1 + *r*_MT_*f*_m_), in which ${R_{{\mathrm{1M}}}}$ is the MT pool longitudinal relaxation rate. ${f_{\mathrm{m}}}$ is obtained from measurement to align with $R_{{\mathrm{ex}}}^{{\mathrm{MT}}}$.

Diverse training data were generated by varying sample parameters *f*_s_, *k*_sw_, *T*_1_, and *T*_2_ for the simulated components, scaling factors *r* for the measured components, and *B*_1_ and *B*_0_ shifts as detailed in supporting information table S6. To extract features from both stroke lesion and normal tissues, two sets of measured components were derived from the mean Z-spectra in the regions of interests (ROIs) of rat brain (#1) as shown in supporting information figure S2. Notably, although the measured components were from a limited sample size, a large and diverse training dataset can still be generated by varying the sample parameters, scaling factors and inhomogeneities. The target guanidine CEST spectrum was obtained using equation (1), where *S*_lab_ corresponds to partially synthetic CEST Z-spectrum with guanidine pool and *S*_ref_ correspond to partially synthetic CEST Z-spectrum without the guanidine pool for their corresponding sample parameters and no *B*_0_ and *B*_1_ shifts. After the training, the network was tested to predict the guanidine CEST effect in four rats (#2–5).

The subtraction of the mfit-poly fitted $R_{{\mathrm{ex}}}^{{\mathrm{amines}}}$ from the six-pool model Lorentzian fitted $R_{{\mathrm{ex}}}^{{\mathrm{amines/guan}}}$ could be another way to quantify $R_{{\mathrm{ex}}}^{{\mathrm{guan}}}$, which can be compared with our ML method. We referred to this approach as Δpoly, and the resulting $R_{{\mathrm{ex}}}^{{\mathrm{guan}}}$, quantified using the AREX metric, was denoted as AREX_Δpoly_.

### 2.6. Generation of tissue mimicking data

Numerical simulations using Bloch-McConnell equations and the same sequence parameters as the MRI experiments were used to generate a set of simulations, termed tissue mimicking data. A seven-pool simulation model consisting of amide at 3.5 ppm, amine at 3 ppm, guanidine at 2 ppm, water, NOE (−1.6), NOE (−3.5), and MT was utilized. The sample parameters along with *B*_1_ and *B*_0_ shifts used for creating this dataset are listed in supporting information table S7. The ground truth guanidine CEST spectra were obtained using equation (1), where the Z-spectra with and without guanidine pool represent *S*_lab_ and *S*_ref_ for the corresponding sample parameters. *B*_0_ and *B*_1_ shifts were excluded for obtaining the ground truth. To create the partially synthetic data, we first selected a single Z-spectrum at random from the tissue-mimicking data and applied a six-pool model Lorentzian fit to that spectrum. The measured components for partially synthetic data were taken from this fit. Next, we generated the partially synthetic data as described in the previous section by varying sample parameters, scaling factors and *B*_0_ and *B*_1_ shifts. The generated data was used to train the ML network to predict guanidine CEST effects. A separate batch of tissue-mimicking data was reserved for testing and results from the ML network were compared to the ground truth to evaluate the accuracy of our method.

### 2.7. Generation of fully synthetic data and preparation of in vivo data for training ML model

To demonstrate the advantage of training on partially synthetic data, the ML network was trained on conventional fully synthetic data and *in vivo* data with limited sample size for comparison. Fully synthetic CEST Z-spectra and their ground truth, were generated using the same methods as for the tissue-mimicking data but varying the sample parameters as listed in supporting information table S8, to account for the variations between synthetic and actual tissues. For training the ML network using *in vivo* data, data from rat brain (#1) acquired at various time intervals (0.5–1 h, 1–1.5 h, 1.5–2 h) were utilized resulting in 1894 voxels. To assess the effectiveness of data augmentation, a technique designed to expand both the size and diversity of the training dataset; in addressing the issue of limited sample size, the dataset was augmented nearly 900-fold. This was achieved by calculating the mean of each pair of Z-spectra derived from the original voxel data. *B*_0_ shifts, similar to those used in generating other datasets, were added by interpolating the *in vivo* Z-spectra. The target guanidine CEST spectrum for the *in vivo* training datasets was derived using voxel-wise PLOF, polynomial fit, Δpoly, the six-pool model Lorentzian fit, and the seven-pool model Lorentzian fit. Each of these methods included *B*_0_ shift correction and was quantified with the AREX metric. The ML-predicted guanidine CEST was termed AREX_ML_.

### 2.8. ML model architecture, training and optimization of frequency offsets

The ML workflow including the architecture of the NN implemented in this study and the process of frequency offset optimization process is illustrated in figure 1(B).

CEST Z-spectral data normalized by *R*_1obs_ were used as inputs for training, while the amplitude (*A*) and width (*W*) of the corresponding guanidine CEST peak were used as targets. The target data can be obtained by replacing Δ*ω* with Δ in the guanidine CEST peak to determine *A* and using the full width at half maximum (FWHM) of the guanidine CEST peak to calculate *W*. The ML-predicted guanidine CEST spectrum was reconstructed using a Lorentzian function with the predicted *A* and *W* values. The NN architecture included an input layer, four dense hidden layers (512, 64, 64, and 16 nodes) and a final output layer with two nodes corresponding to *A* and *W*. ReLU activation was applied to all layers except the final output layer. The model was optimized using the Huber loss function and the Adam optimizer (torch.optim.Adam), with a learning rate of 1 × 10^−3^ and a batch size of 64. The Huber Loss function was chosen to balance the benefits of mean squared error and mean absolute error (MAE), thereby reducing the impact of outlier influence. Training was conducted for approximately 25 epochs with early stopping implemented to prevent overfitting. The NN was trained on thirteen different datasets as shown in supporting information table S9. For model trained on partially synthetic data and fully synthetic data, Gaussian noise with a standard deviation of 0.01 was added to the data to simulate real tissue signals. A description of all the comparisons performed in this study is given in supporting information table S9.

To identify the most informative frequency offsets for predicting the guanidine CEST effect, we applied a gradient-based feature attribution method that directly leverages the trained prediction network. The network was first trained using the tissue mimicking simulated dataset containing the full set of frequency offsets, which served as a common reference dataset for all ML approaches. The model was trained using the same procedure described in the previous section, with the Huber loss used to minimize the prediction error. After training, we computed the gradient of the network’s output with respect to each input feature across the entire training set. An average of the absolute gradient across all training samples was computed for each feature, producing a vector of gradient magnitude (g) that quantifies the sensitivity of the model output to each frequency offset. The corresponding gradient profile is shown in supporting information figure S3. A selection threshold was defined as the mean gradient magnitude across all frequency offsets, as this criterion is simple and highly interpretable. Frequency offsets with gradient magnitudes exceeding this threshold were retained, as they exert a stronger influence on the network’s predictions. Unlike the recursive feature elimination (Viswanathan *et al* 2025a) technique used previously to optimize the frequency offsets, which relies on a separate surrogate model for feature ranking, this gradient-based approach uses the same trained network to identify important features. This ensures that the selected frequency offsets are more closely aligned with the model’s learned representation and are more sensitive to input changes captured by the network. The network was then retrained using this refined subset of features, resulting in a more focused and biologically meaningful model.

To assess the robustness of our optimization, the network was trained using partially synthetic data with measured components derived from the tissue-mimicking data with varying levels of Gaussian noises (*σ* = 0.02, 0.01, 0.0067, and 0.005). The model was then tested using Monte Carlo simulations. One thousand data samples were generated at each noise level, to determine the resulting coefficient of variance in the predicted guanidine CEST amplitude. Considering that the acquisition time after the optimization (140 s) is 1/3.5th of the time required without the optimization (490 s), the standard deviation in the predicted guanidine amplitude with the optimization is divided by 1.87.

The model was implemented in Python using PyTorch. Training times varied depending on the data: models trained on partially and fully synthetic datasets requiring around 2 h, while *in vivo* data without augmentation took about 4 mins. The inference for generating a guanidine CEST map for a rat brain took approximately 1.6 s on a Dell system with a 12th Gen Intel(R) Core (TM) i7-12700 2.10 GHz processor.

### 2.9. Data analysis and statistics

The criterion used for assessing model accuracy was the MAE between the predicted guanidine CEST spectra and the ground truth spectra, with the frequency range from 1.5 ppm to 2.5 ppm. Similar assessments were performed on the guanidine CEST spectra obtained using the PLOF, polynomial fit, Δpoly, six-pool model Lorentzian fitting, and seven-pool model Lorentzian fit. This comparison allowed for a systematic evaluation of whether the ML approach leveraging partially synthetic data outperformed the conventional methods.

ROIs for both the stroke lesion and the corresponding contralateral normal tissue were defined based on the lesion structures observed on the *T*_1obs_ map at different time intervals. The Wilcoxon rank sum test was used to assess the differences in parameters between stroke lesions (L) and contralateral normal tissues (N) and were deemed statistically significant if *p*-value was less than 0.05.

## 3. Results

### 3.1. Optimization of frequency offsets

**Figure 2.**
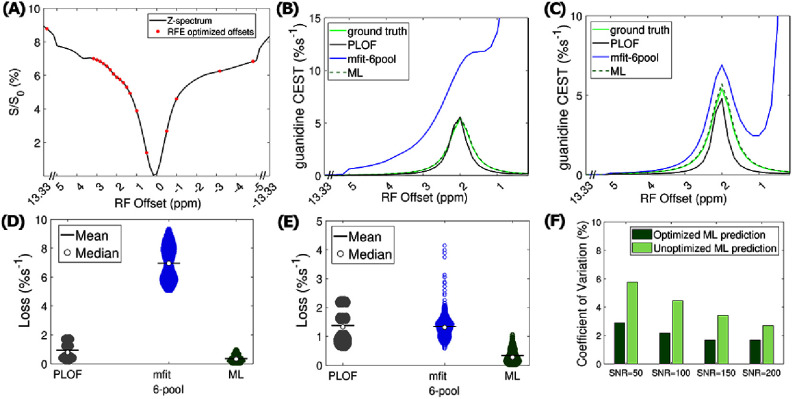
(A) A representative Z-spectrum from the tissue-mimicking testing data, along with the optimized frequency offsets (red dots). (B) Corresponding guanidine CEST spectra quantified by AREX_PLOF_, AREX_mfit_6pool_, and AREX_ML_ using partially synthetic data without optimization and (C) with optimization. The ground truth guanidine CEST spectrum is also shown in (B), (C) for reference. (D), (E) MAE comparisons among the methods in (B), (C) for all test samples. (F) Comparison of the coefficient of variation of ML-predicted guanidine CEST amplitudes from the Monte Carlo simulations, with and without optimization, across various noise levels. Comparisons with other methods are provided in the supporting figures due to space constraints.

Out of the initial 69 frequency offsets, the model identified 19 important offsets, listed in Supporting information table S10. The selected optimized frequency offsets, shown in figure 2(A), appear to capture the features from amines/guan, water, and MT peaks.

### 3.2. Validation using tissue mimicking data

Figure 2(A) shows a representative Z-spectrum along with the optimized acquisition points. Figures 2(B) and (C), as well as supporting information figures S4(B) and (C) compare the guanidine CEST spectra quantified by various methods—PLOF, polynomial fit, Δpoly, multiple-pool model Lorentzian fit (six-pool and seven-pool models), and ML using partially synthetic data—against the corresponding ground truth, without and with the optimization, respectively. It is evident that the ML-predicted guanidine CEST spectrum closely matches the ground truth, both with and without optimization.

Additionally, the PLOF-quantified guanidine CEST spectrum closely aligns with the ground truth only in the absence of optimization. In contrast, other methods exhibit significant deviations from the ground truth. Figure 2(D) and supporting information figure S4(D) present a comparative analysis of the loss between the ground truth and these methods across all test samples, without optimization while figure 2(e) and supporting information figure S4(E) show the analysis with optimization. The ML network achieves an MAE of 0.33%s^−1^ without optimization and 0.36%s^−1^ with optimization, for all test samples, indicating that it predicts guanidine CEST effect with high accuracy.

In contrast, the six-pool and seven-pool Lorentzian fit exhibit much higher average MAEs of 6.97%s^−1^ and 2.44%s^−1^ without optimization, and 1.34%s^−1^ and 2.24%s^−1^ with optimization, respectively. PLOF performs well using the Z-spectrum without optimization, with an average MAE of 0.94%s^−1^, but shows larger deviation from the ground truth when optimization is applied, resulting in an average MAE of 1.86%s^−1^. A similar trend is observed for polynomial fit and Δpoly, with MAE values of 1.78%s^−1^ and 1.99%s^−1^, respectively, for fitting without optimization. However, the inclusion of optimization increases their MAE values to 7.21%s^−1^ and 9.79%s^−1^, respectively. Supporting information figures S5 and S6 compare the losses between PLOF, mfit_6pool, additional traditional methods and our ML method where the measured components were derived from the multiple-pool model Lorentzian fit of nine other Z-spectra selected at random, as well as the averaged Z-spectra from 900 and 1800 samples randomly selected within the tissue-mimicking data. The results align consistently with those shown in figures 2(D) and (E). Figure 2(F) compares the coefficient of variation of the predicted guanidine CEST amplitudes with and without optimization from the Monte Carlo simulations, across a range of noise levels. The SNR efficiency improved significantly after optimization.

### 3.3. Application in MCAO stroke animal model

**Figure 3.**
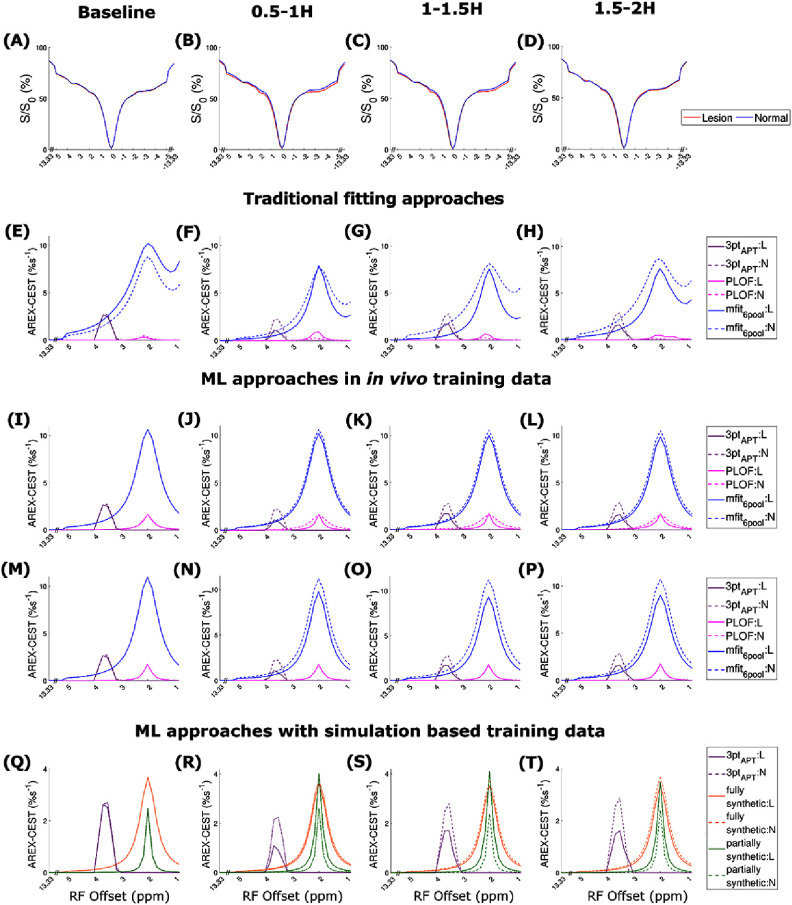
Comparison of the mean CEST Z-spectra (A)–(D) and the mean guanidine CEST spectra between the stroke lesion (L) and contralateral normal tissues (N), in the four testing rats (#2–5), quantified using various methods (E)–(T), acquired before (baseline) and at a few time points (0.5–1 h, 1–1.5 h, and 1.5–2 h) after the stroke onset. The guanidine CEST spectra were quantified using the following methods: (E)–(H) PLOF and six-pool model Lorentzian fit (mfit_6pool); and an ML network trained on: (I)–(L) *in vivo* data with training targets derived from the PLOF and mfit_6pool, (M)–(P) augmented (aug) *in vivo* data with training targets from various methods, and (Q)–(T) fully synthetic data and partially synthetic data. The APT spectra, quantified by the three-point method, were plotted in (E)–(T) for comparison. Z-spectra with optimized offsets were used in all methods to quantify the guanidine CEST effect. Comparisons with other methods are provided in the supporting figures due to space constraints.

Figure 3 compares the mean CEST Z-spectra and the mean guanidine CEST spectra between the lesion and normal tissues, quantified using various methods, along with optimization. The plots with standard deviation for the data presented in figure 3 along with additional methods are provided in supporting information figure S7. The results presented in figure 3 along with additional methods, without optimization, are shown in supporting information figure S8. There is a notable increase in the CEST effect at 2 ppm in the Z-spectra from the lesion after stroke onset, demonstrating the sensitivity of the guanidine CEST effect to stroke. Additionally, the CEST effect at 2 ppm in the Z-spectra from the lesion is more pronounced than the APT effect and appears as a narrow peak.

The PLOF, polynomial fit, and Δpoly methods provide a peak at 2 ppm with optimization, however, the PLOF method results in a very small peak, while the polynomial fit and Δpoly produce a large and negative peak, consistent with the simulations shown in figures 2(B) and (C), supporting information figures S4(B) and (C). This discrepancy may be due to insufficient data available after optimization for these methods to accurately determine *S*_ref_. The six-pool model Lorentzian fit produces a broad and large peak at 2 ppm, while the seven-pool model Lorentzian fit results in a very small peak at 2 ppm, consistent with the simulations. This confirms that the six-pool model Lorentzian fit captures contributions from both the broad amine CEST effect and the guanidine CEST effect. In contrast, the seven-pool model Lorentzian fit is unable to accurately isolate the guanidine CEST effect from the amine CEST effect, leading to an underestimation of the guanidine contribution. The guanidine CEST spectra predicted by the ML network with training on in vivo data and training targets derived from various methods generally deviate from the corresponding training targets, even with data augmentation, suggesting that training on limited sample size or with insufficient data features is unreliable. The guanidine CEST spectra predicted by the ML network trained on fully synthetic data exhibit a broad peak. This may be due to the presence of multiple unknown amine pools or downfield aromatic NOE effects (Zhou et al 2023), and the absence of sufficient simulation models in the fully synthetic data, leading the ML network to incorrectly assign these signals to guanidine. In contrast, in the partially synthetic data, these signals were attributed to the fitted $R_{{\mathrm{ex}}}^{{\mathrm{amines}}}$. In figures 3(Q)–(T), the guanidine CEST effect using the ML network with training done on partially synthetic data was much higher than the APT effect in stroke lesions, demonstrating the enhanced signal intensity in lesions using guanidine CEST imaging.

**Figure 4.**
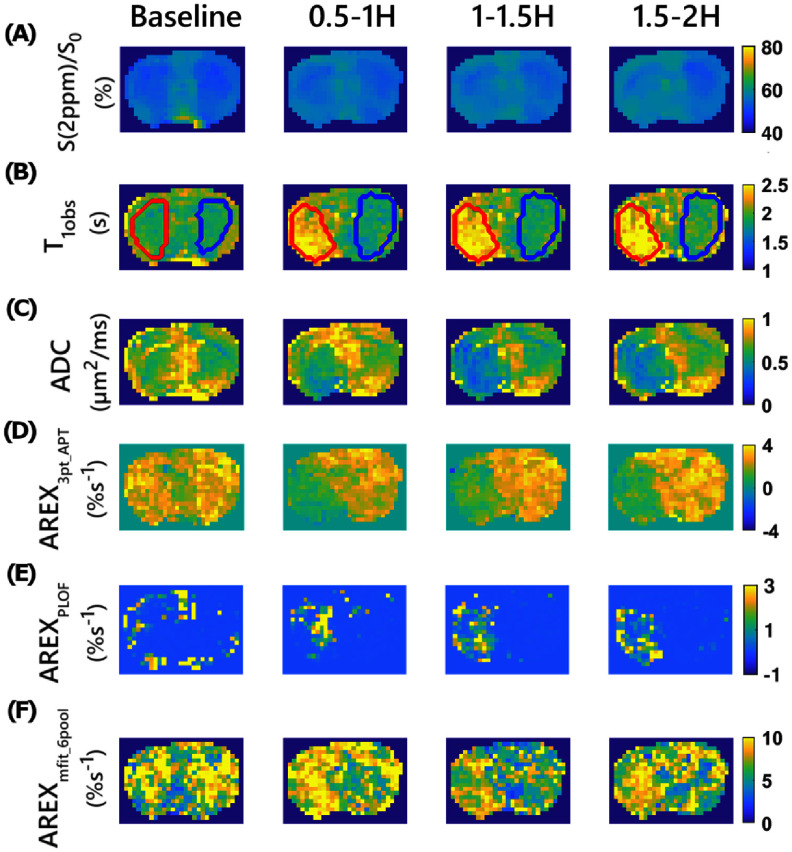
Maps of (A) Z-spectral image at 2 ppm, (B) *T*_1obs_, (C) ADC, (D) AREX_3pt_APT_, (E) AREX_PLOF_ and (F) AREX_mfit_6pool_ acquired at baseline and few time points after stroke onset (0.5–1 h, 1–1.5 h, and 1.5–2 h) from testing rat brain (#2). Z-spectra with optimized offsets were used in all methods to quantify the guanidine CEST effect. The ROIs of stroke lesion (red) and contralateral normal tissue (blue) are also shown in the *T*_1obs_ map. Negative values and zero values were found by using some methods, demonstrating their lack of robustness. The color bar is adjusted to reflect these limitations by displaying zero and negative values. Comparisons with other methods are provided in the supporting figures due to space constraints.

**Figure 5.**
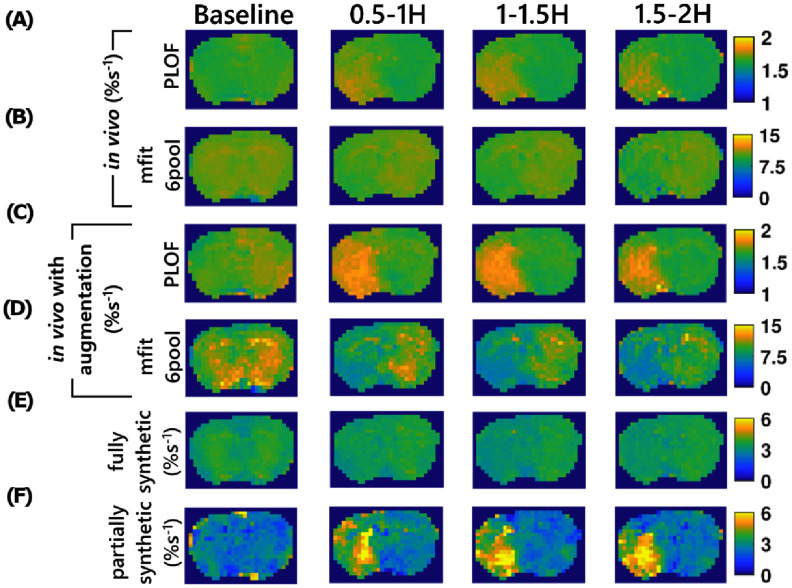
Maps of AREX_ML_ with the ML network trained on *in vivo* data with training targets from (A), (D) PLOF, (B), (G) mfit_6pool without and with data augmentation, as well as on (E) fully synthetic data and (F) partially synthetic data acquired at baseline and few time points after stroke onset (0.5–1 h, 1–1.5 h, and 1.5–2 h) from testing rat brain (#2). Z-spectra with optimized offsets were used in all methods to quantify the guanidine CEST effect. Comparisons with other methods are provided in the supporting figures due to space constraints.

Figure 4 presents a comparison of Z-spectral image at 2 ppm, *T*_1obs_ map, ADC map, APT map quantified using the three-point method, and guanidine CEST maps quantified using PLOF and six-pool model Lorentzian fit along with optimization, from a representative rat. Figure 5 shows the maps of the predicted guanidine CEST effect using ML networks trained with *in vivo* data, whose training targets were derived from quantification methods mentioned in figure 4, fully synthetic data, and partially synthetic data, all with optimization, from a representative rat.

The results from all rats along with the additional methods obtained using optimized offsets are shown in supporting information figures S9–S16 and results without optimization are shown in figures S17–S24. Hypointense signals were consistently observed in the left hemisphere of the brain on the ADC and APT maps in all rats, confirming the successful induction of stroke. The Z-spectral image at 2 ppm showed weak and ambiguous variations in the left hemisphere, indicating the presence of confounding effects and underscoring the need to isolate the guanidine CEST effect. PLOF, polynomial fit, and Δpoly methods show hyperintense signals in the lesion without optimization but produce poor image quality with optimization. The six-pool model and seven-pool model Lorentzian fits show no clear contrast between the lesion and normal tissues without optimization. However, the seven pool model Lorentzian fit shows hypointense signal with optimization. Training the ML network using *in vivo* data with training targets from different quantification methods, both without and with data augmentation, shows either no clear contrast or only contrast within a very narrow range, likely due to the limited sample size or insufficient data features. When trained with fully synthetic data, the network provided contrast without optimization but fails to do so with optimization. In contrast, when trained on partially synthetic data, the network shows clear contrast both with and without optimization.

**Figure 6.**
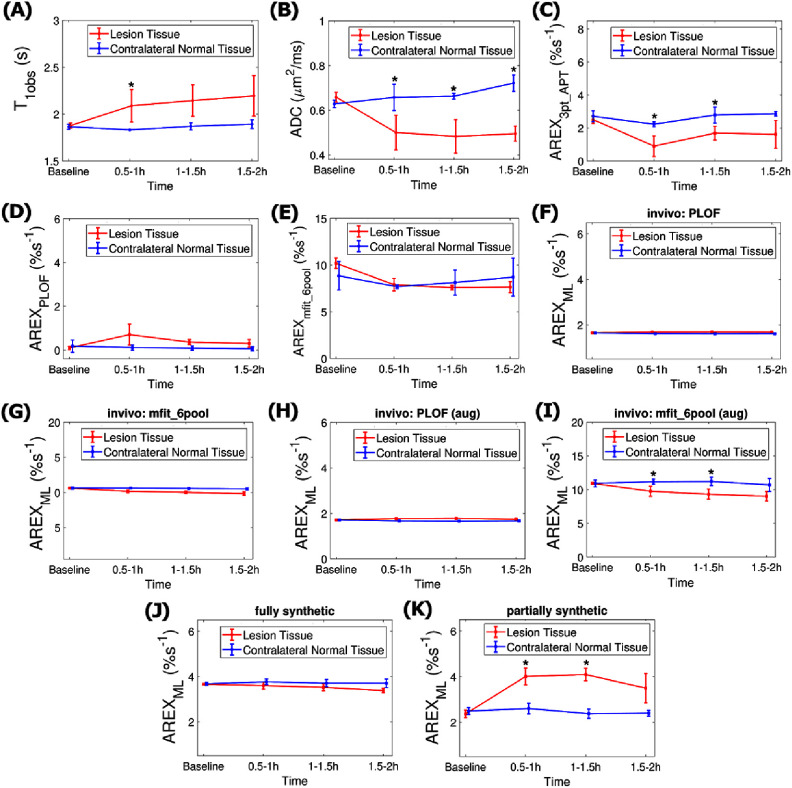
Time-dependent statistics (A) *T*_1obs_, (B) ADC, (C) AREX_3pt_APT_, (D) AREX_PLOF_, (E) AREX_mfit_6pool_, AREX_ML_ values with the ML network trained on: (F,G) *in vivo* data with training targets from PLOF and mfit_6pool, (H,I) *in vivo* data with training targets from PLOF and mfit_6pool with data augmentation, (J,K) fully synthetic data and partially synthetic data, acquired at baseline and few time points after stroke of onset (0.5–1 h, 1–1.5 h, and 1.5–2 h) in stroke lesion (red) and contralateral normal tissue (blue). (* *P* < 0.05) Z-spectra with optimized offsets were used in all methods to quantify the guanidine CEST effect. Comparisons with other methods are provided in the supporting figures due to space constraints.

Figure 6 shows the statistics of the parameters presented in figures 4 and 5 across all time intervals. The statistics for results in figure 6 along with additional methods obtained using optimized frequency offset are shown in figure S25 and without optimization are shown in supporting information figure S26.

Notably, besides the ADC and APT, the guanidine CEST predicted by the ML network trained on partially synthetic data, both with and without optimization, shows a significant increase in the lesion at 0.5–1 h and 1–1.5 h after stroke onset. In contrast, the guanidine CEST predicted by the ML network when trained with fully synthetic data shows these changes only without optimization, indicating that the scan time for this method cannot be reduced.

Additionally, the PLOF, polynomial fit, and Δpoly methods exhibit a significant increase in the lesion at 1–1.5 h after stroke onset without optimization; however, their standard deviations are much higher than those of the ML network trained on partially synthetic data, highlighting the increased robustness of our ML method. With optimization, both the PLOF and polynomial fit methods show no significant changes. The Δpoly method shows significant increase in the lesion at 1–1.5 h after stroke onset with optimization. However, their negative values are unexpected. Furthermore, when training the ML network on *in vivo* data using training targets from various methods except for the six-pool model Lorentzian fit, shows no significant variations at 0.5–1 h and 1–1.5 h after stroke onset. The six-pool model Lorentzian fit shows a significantly decreased signal in the lesion, which is unexpected. This can be explained by the contributions from both amines and guanidine. In figures 3(E)–(H), the six-pool model Lorentzian fitted peak includes a narrow peak at 2 ppm superimposed on a broader amine CEST peak. Although the narrow guanidine CEST signal increases, the broad amine CEST signal decreases within the lesion, leading to a slight reduction in the mixed signal at 2 ppm. The mechanism underlying the diminished amine CEST signal remains unclear and warrants further investigation.

**Figure 7.**
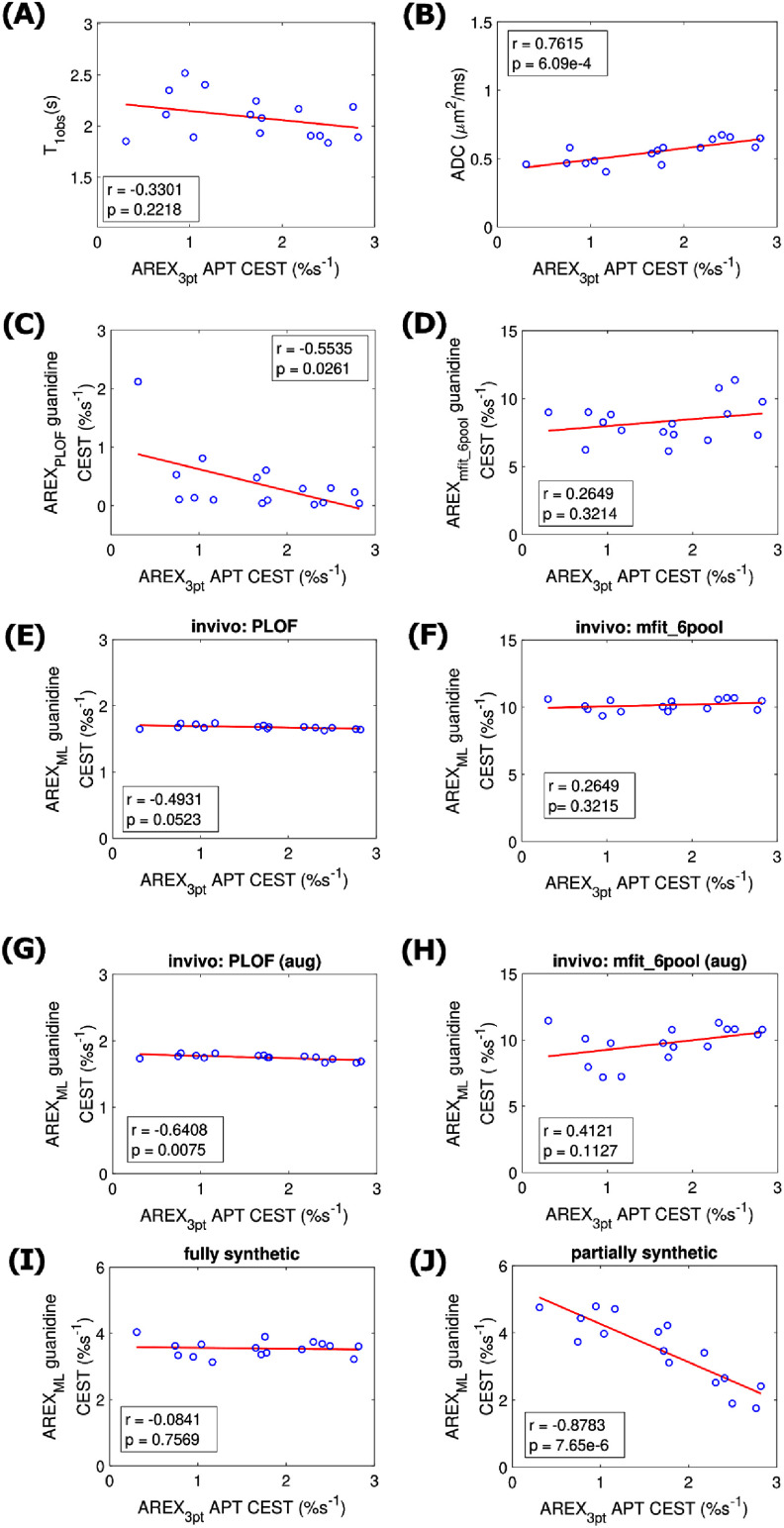
Correlations between the AREX_3pt_APT_ and (A) *T*_1obs_, (B) ADC, (C) AREX_PLOF_, (D) AREX_mfit_6pool_, AREX_ML_ values with the ML network trained on: (E), (F) *in vivo* data with training targets from PLOF and mfit_6pool, (G), (H) *in vivo* data with training targets from PLOF and mfit_6pool with data augmentation, (I), (J) fully synthetic data and partially synthetic data. Z-spectra with optimized offsets were used in all methods to quantify the guanidine CEST effect. The circles represent the mean values of each ROI from stroke lesion acquired at different time points after onset of stroke. Spearman’s rank correlation coefficient (r) and p value of the correlation are provided. The solid line represents the linear regression of all data points. Comparisons with other methods are provided in the supporting figures due to space constraints.

Figure 7 shows the correlations between the APT effect, quantified by the three-point method, and the guanidine CEST effect, quantified by various methods with optimization in stroke lesion. The correlations for the results in figure 7 along with additional methods obtained using optimized frequency offset are shown in figure S27 and without optimization are shown in supporting information figure S28.

The PLOF, Δpoly, ML network trained on *in vivo* data with training target from the PLOF method and with augmentation, and the ML network trained on partially synthetic data show significant correlations, both with and without optimization. The polynomial fit and the ML network trained on fully synthetic data show significant correlations only without optimization. Notably, the ML network trained on partially synthetic data exhibits the smallest p-values among all the methods when optimization is applied. Supporting information figures S29 and S30 illustrate the correlations between the guanidine CEST effect predicted by the ML network trained on partially synthetic data and that quantified by various other methods, with and without optimization, respectively, in stroke lesion. Both Δpoly and ML network trained on *in vivo* data with training target from the PLOF method with augmentation show significant correlations, both with and without optimization. The PLOF method shows significant correlation only with optimization.

## 4. Discussion

When training our ML network on partially synthetic data, it demonstrates greater accuracy than all traditional fitting methods in quantifying the guanidine CEST effect. Additionally, it outperforms both traditional methods and ML networks trained on other types of data, in highlighting lesion contrast in the animal stroke model. After optimization, the total scan can be reduced to 19 sampling points plus a control scan, which totals approximately 2.3 min. This efficiency makes it a feasible and practical method for detecting ischemic stroke. Furthermore, the strong negative correlation between the guanidine CEST effect predicted by our ML network and the APT effect confirms its sensitivity to pH changes. Compared with APT, the guanidine CEST effect exhibits hyperintense contrast and higher signal intensity in the stroke lesions. This makes it a more effective imaging method for identifying and characterizing ischemic stroke.

The guanidine CEST effect operates through a different mechanism than the APT effect in assessing pH variation in ischemic stroke. The APT effect arises solely from proteins and peptides. Since the protein/peptide content in ischemic stroke may not change significantly, the APT effect primarily reflects the variation in the amide–water exchange rate which is pH-dependent. In contrast, the guanidine CEST effect includes contributions from both protein arginine and creatine. Although creatine concentration may slightly change due to disrupted energy metabolism during stroke (Zong *et al* 2014), potentially reducing the sensitivity of the guanidine CEST effect to the guanidine-water exchange rate and thus pH, the strong correlation between the guanidine CEST effect and the APT effect in stroke lesions suggests that such fluctuations in creatine concentration have a negligible impact on pH assessment in this animal model. Moreover, a previous approach (Jin *et al* 2017) that involves subtracting the APT effect from the guanidine CEST effect to enhance pH sensitivity underscores the strong association between the guanidine CEST effect and pH. This is likely due to the relatively lower contribution of creatine compared to protein arginine in the brain (Chen *et al* 2017, Zhang *et al* 2017b, 2023, Xu *et al* 2023). In other animal models or human stroke patients, validation of the sensitivity of the guanidine CEST to pH is warranted. Further quantification of the underlying exchange rate could reduce the influence from the varied pool concentration, thereby enhancing pathological relevance. However, this typically requires multiple saturation powers and high SNR, which can extend scan times. Future research should explore the feasibility of applying the creatine exchange rate in acute stroke.

This study applied a relatively low saturation field of 1 *μ*T. At higher saturation fields, the guanidine CEST effect may be more pronounced, leading to higher signal intensity. However, the dependence on the exchange rate may be reversed; the guanidine CEST effect could decrease with a reduced guanidine-water exchange rate, as shown in supporting information figure S1. This is also evident in a previous CEST imaging study conducted with a saturation field of 2 *μ*T (Heo *et al* 2017). Further studies are required to optimize the *B*_1_ value for guanidine CEST imaging in ischemic stroke.

The relatively high loss observed in the multiple-pool model Lorentzian fitted and Δpoly fitted $R_{{\mathrm{ex}}}^{{\mathrm{guan}}}$ suggests that the measured components, including $R_{{\mathrm{ex}}}^{{\mathrm{MT}}}$ and $R_{{\mathrm{ex}}}^{{\mathrm{amines}}}$ derived from these methods, may also exhibit significant errors in their amplitudes. However, we found that these amplitude errors do not adversely affect our ML predictions. This is because we only utilize the line shape information of the measured components, and apply a scaling factor to adjust their amplitudes, to generate the partially synthetic training data. Supporting information figure S31 shows that although the MAE values for the multiple-pool model Lorentzian fitted $R_{{\mathrm{ex}}}^{{\mathrm{MT}}}$ and the mfit-poly fitted $R_{{\mathrm{ex}}}^{{\mathrm{amines}}}$—which reflect the amplitude fitting errors—are high compared to our ML predictions using partially synthetic data, the correlation between the fitted $R_{{\mathrm{ex}}}^{{\mathrm{MT}}}$ and $R_{{\mathrm{ex}}}^{{\mathrm{amines}}}$ spectra and their ground truth spectra between 1.5 ppm and 5 ppm are very strong (Pearson correlation coefficient >0.999 and 0.995, respectively). This indicates a high similarity of the line shapes between the fitted and ground truth spectra, suggesting that using line shape information can mitigate the influence of fitting errors introduced by the multiple-pool Lorentzian fit and mfit-poly methods. The $R_{{\mathrm{ex}}}^{{\mathrm{amines}}}$ spectrum between 0 ppm and 1.5 ppm deviates significantly from its ground truth. However, errors in the fitted $R_{{\mathrm{ex}}}^{{\mathrm{amines}}}$ within this frequency range, which is close to water, can be compensated by *R*_eff_ when they are used to reconstruct the CEST Z-spectra. Additionally, the optimized sampling points shown in figure 2(A) suggest that the accuracy of the predicted guanidine CEST effect mainly relies on CEST signals beyond 1.5 ppm. The lower loss observed in ML predictions using partially synthetic data, compared to other methods, demonstrates the effectiveness of our approach.

As shown in our previous work (Viswanathan *et al* 2025a), the line shape information derived from measured components plays an important role in capturing the intrinsic spectral diversity of CEST data. Fully synthetic datasets generated purely from Bloch McConnell simulations may not adequately represent these complex spectral features due to simplifications in the underlying models or bias in the parameter selection. In contrast, partially synthetic datasets that incorporate experimentally measured components preserve more realistic line shape information, enabling improved prediction accuracy and physiological relevance. In our prior study, we observed that incorporating measured components from both normal and ischemic tissues provided the most accurate representation of spectral heterogeneity. However, models trained using measured components from either tissue type alone also achieved comparable performance, indicating that differences in line shape between tissue types contribute minimally to prediction variability.

Compared to ML networks trained on *in vivo* data for clinical applications, our method’s ability to effectively use a limited sample size for training is critical. Successful training of ML networks on *in vivo* data typically requires a large volume of samples. However, acquiring sufficient and high-quality *in vivo* CEST data in stroke patients is challenging due to the urgent need for immediate treatment, which leaves a limited time window for data acquisition.

Our platform is based on a steady-state CW-CEST signal model. However, in human imaging, pulsed-CEST sequences are commonly used. This issue can be addressed by utilizing a previously derived analytical solution for pulsed saturation, which approximates the pulsed-CEST signal as repeated hard pulses (Gochberg *et al* 2018). On some clinical scanners equipped with multi-channel RF amplifiers that enable pseudo-continuous RF saturation, our method can be directly applied (Keupp *et al* 2011, Wada *et al* 2015, Zhou *et al* 2022). Furthermore, non-steady-state CEST imaging is typically employed in human imaging to reduce total scan time. This can be addressed by using either perturbation of longitudinal relaxation rate in the rotating frame analysis (Wang *et al* 2017) or quasi-steady-state (QUASS) analysis (Sun 2021) to convert non-steady-state CEST signals into steady-state CEST signals. It has been reported that a super-Lorentzian line shape is more suitable for modeling *in vivo* tissue MT effects (Morrison *et al* 1995). In future studies, this line shape could be utilized in the fitting process and integrated into the generation of partially synthetic data to evaluate whether it enhances accuracy further.

## 5. Conclusion

In this study, we trained an ML network using partially synthetic data to quantify the guanidine CEST effect in ischemic stroke. This approach not only delivers accurate and precise quantification of guanidine CEST effect but also reduces CEST scan time by 72% compared to conventional CEST acquisitions. The ML-enhanced guanidine CEST imaging provides a pathway for rapid pH-sensitive CEST imaging for stroke assessment, offering hyperintense contrast and higher signal intensity in stroke lesions compared to the traditional APT imaging method.

## Data Availability

All raw data and source code for processing and analysis are available on our lab’s GitHub: https://github.com/CESTlabZu/ML_Guanidine_Stroke (Viswanathan *et al* 2025b). Supporting Information Document available at http://doi.org/10.1088/1361-6560/ae4167/data1.
